# Multi-Scale Analyses and Modeling of Metallic Nano-Layers

**DOI:** 10.3390/ma14020450

**Published:** 2021-01-18

**Authors:** Zara Moleinia, David F. Bahr

**Affiliations:** School of Materials Engineering, Purdue University, West Lafayette, IN 47907, USA; dfbahr@purdue.edu

**Keywords:** multi-scale, metallic nano-layer, crystal plasticity, deep-learning, single layer calibration, homogenization, temperature effects

## Abstract

The current work centers on multi-scale approaches to simulate and predict metallic nano-layers’ thermomechanical responses in crystal plasticity large deformation finite element platforms. The study is divided into two major scales: nano- and homogenized levels where Cu/Nb nano-layers are designated as case studies. At the nano-scale, a size-dependent constitutive model based on entropic kinetics is developed. A deep-learning adaptive boosting technique named single layer calibration is established to acquire associated constitutive parameters through a single process applicable to a broad range of setups entirely different from those of the calibration. The model is validated through experimental data with solid agreement followed by the behavioral predictions of multiple cases regarding size, loading pattern, layer type, and geometrical combination effects for which the performances are discussed. At the homogenized scale, founded on statistical analyses of microcanonical ensembles, a homogenized crystal plasticity-based constitutive model is developed with the aim of expediting while retaining the accuracy of computational processes. Accordingly, effective constitutive functionals are realized where the associated constants are obtained via metaheuristic genetic algorithms. The model is favorably verified with nano-scale data while accelerating the computational processes by several orders of magnitude. Ultimately, a temperature-dependent homogenized constitutive model is developed where the effective constitutive functionals along with the associated constants are determined. The model is validated by experimental data with which multiple demonstrations of temperature effects are assessed and analyzed.

## 1. Introduction

Crystalline nano-layers are formed by alternating nanoscale metallic lamellae where the reduction of size to the order of nanometers instigates physicochemical attributes notably departing from those of the bulk counterparts. Metallic nano-systems exhibit exceptional mechanical properties in which the layer size is comparable with the electron or phonon mean free path [1]. Size effects are pivotal aspects in copiously modulated thermodynamically non-equilibrium metallic nano-composites where the rigid body relaxation is limited due to the constraints of neighboring nano-phases [2]. The salient, differentiating traits in mechanical responses are assigned to the primary role of layer thicknesses and significant density of interfaces. These features are considered the controlling parameters to modify and modulate the strength and multi-functionality of metallic nano-structures [3] where the dynamic characteristics of the atomic energy with local non-equilibrium multi-valley potentials [4,5] promote the performances of nano-metals to be governed and altered towards desired applications.

Typically, interfaces function as sources, sinks, and barriers for defects, particularly dislocations. Dislocations, with anisotropic mobility and spreading cores throughout interfaces, describe various aspects of metallic nano-layers physical properties [6,7] and represent them as tunable structures with remarkable responses in extreme environments [8,9]. Interfaces of distinct atomic structures supply glissile dislocations into contiguous building blocks by virtue of inelastic deformation. The reduction of activation volume induces dislocation mechanisms at a shorter distance [10,11] where the amplified emission of interface dislocations and the onset of plasticity through interphases are of consequence.

The inverse dependence of strength on grain size refinement in metals and metallic alloys with an average size in the order of micrometers follows the Hall–Petch relation [12,13] emphasizing on deformation kinematics rooted from dislocation pile-up against interfaces along with other transgranular dislocation mechanisms. This quality remains intact regardless of synthesizing approaches such as physical vapor deposition (PVD) [14,15] or accumulative roll bonding (ARB) [16]. However, once the average grain size is reduced to the orders of nanometers evincing the participation of fewer dislocations in pile-up, the inadequacy of this relation conceivably emerges in a reduced Hall–Petch slope. Instead, it is governed by the Orowan mechanism [17] especially pronounced at heterophase boundaries [18].

Hereby, a more detailed governing relation including the main features of size and constituent effects must be accommodated to analyze metallic nanolamellars responses at nano-regions. In general, these types of models deliver verifiable, solid results, however, with complex, nonlinear structures, hence, the elevated cost of computational processes. Consequently, multi-scale analyses are sought as proper resolutions especially when nonlinearity is involved [19]. In circumstances where size effects are crucial to final assessments, homogenized ensembles inherently possess the governing elements through the variation of the fundamental thermodynamics behaviors including internal energy and entropy that are intrinsically nonlinear and can be statistically generalized on a larger scale. In this sense, capturing temperature effects by homogenization approaches is a significant advantage considering limited experimental results due to a variety of encountered challenges throughout testing. These difficulties include rigorously controlling the atmosphere under which tests are performed to prevent specimens’ chemical and microstructural alterations, precise management of thermal gradients between the sample and fixture during the process to avoid thermally misfit deformation and noise in the load and displacement sensors drifting the results, and challenges as such [20,21].

This work centers on constructing a robust theoretical approach while alleviating computational encumbrance through curtailing partly dependent phenomena into optimized independent variables. The crystal plasticity finite element (CPFE) approach in the large deformation platform is utilized due to its high capacity of analyzing anisotropic nature of crystalline materials, grain interactions, interface abrupt mechanical transitions, mixed deformation mechanisms, complex boundary conditions, and diverse phenomenological and physics-based constitutive models [22,23].

The multi-scale computational schemes are pursued leading to the development of multiple governing relations. At the nano-scale, a size-dependent constitutive model and a deep-learning approach named the single layer calibration (SLC) method with the ability to obtain generalized parameters applicable to a broad range of setups are developed. These models simulate, predict, and design the responses of metallic nano-layers in the range of 20 nm to 1 μm with any arbitrary geometrical combinations through a single process. A homogenized crystal plasticity-based model is established with the rendition of the nano-structural critical features. The effective functionals are realized for which the associated relations and parameters obtained by way of metaheuristic genetic algorithms. The model is designed consistent with the nano-model backbone remarkably conducive in simulations of polycrystalline microstructures and significantly expediting computational processes by several (>4) orders of magnitude, while retaining accuracy. Ultimately, a temperature-dependent constitutive model is developed to determine the effects of temperature on the overall responses of metallic nanolamellars. The validation of nano- and homogenized models proceeds through the simulation of several copper-niobium, Cu/Nb, nano-layers compared with experimental data. Accordingly, at each spatial spectrum, multiple predictive case studies are assessed and discussed revealing the impacts of size, loading patterns, layer type, geometrical combination, elevated temperature, and process speed on the overall thermomechanical responses of metallic nano-composites.

All the segments including the developed nano-scale size-dependent constitutive model, the deep-learning single layer calibration method, homogenized constitutive model, temperature-dependent constitutive model, genetic algorithms, numerical solvers, and process optimizers are implemented through three-dimensional crystal plasticity nonlinear finite element codes in the large deformation platform. A dedicated cluster has been constructed with specific architecture and orchestration policies compatible with the current data processing and workloads.

## 2. Materials and Methods

Metallic nano-layers are investigated through crystal plasticity large deformation finite element platforms to analyze and predict the associated thermomechanical responses. Deformations up to 10% strain are utilized based on the existing experimental data for the purpose of training the models, though it is possible to proceed the simulation for further values. An advanced multi-scale approach is established to expedite computational procedures while the precision is maintained. Thereby, diverse theoretical domains including plastic deformation kinematics, entropic kinetics, and statistical mechanics of a system at two nano- and homogenized level are employed in order to acquire proper constitutive models addressing the main features of size effects in these types of materials. Subsequently, the parameters in the developed constitutive models are calibrated and determined through a deep-learning method.

### 2.1. Plastic Deformation Kinematics

Finite strain kinematics are accommodated through a multiplicative decomposition of total deformation gradient, F, into the elastic, $F^{e}$, and plastic, $F^{p}$, parts as $F=F^{e}F^{p}$. The rate dependence is manifested in the rate of the deformation gradient, $\dot{F}$, through the velocity gradient, $l=\dot{F}F^{-1}$. The plastic velocity gradient, $l^{p}=\dot{F^{p}}F^{-p}$, involving plastic shear strain rate, ${\dot{\gamma }}^{\alpha }$, on the slip systems, α, [24,25], specified with $l^{p}=\sum_{\alpha =1}^{N}{\dot{\gamma }}^{\alpha }s_{0}^{\alpha }$, are utilized to solve the indeterminate equation of $F=F^{e}F^{p}$, where $s_{0}^{\alpha }=m_{0}^{\alpha }⊗n_{0}^{\alpha }$ is the Schmid tensor in which $m_{0}^{\alpha }$ and $n_{0}^{\alpha }$ are the slip direction and normal, respectively.

The plastic shear strain rate for each slip system is calculated through the Orowan equation [26], ${\dot{\gamma }}^{\alpha }=\rho_{m}^{\alpha }\;b\;v^{\alpha }$, where $\rho_{m}^{\alpha }$ is the mobile dislocation density, *b* the Burgers vector, and $v^{\alpha }$ the average mobile dislocation velocity. The integration of $\dot{F^{p}}$ determines $F^{p}$ and, accordingly, $F^{e}$ through $F^{e}=FF^{-p}$ which yields the second Piola–Kirchhoff stress, S, in terms of Lagrangian strain, $E=\frac{1}{2}\left(F^{eT}F^{e}-I\right)$, and the anisotropic material elastic tensor, C, with S=CE. Solving the equilibrium equation in the current configuration requires the calculation of Cauchy stress, $\sigma =\frac{1}{|F^{e}|}F^{e}SF^{eT}$, and its derivative with respect to true strain to obtain the system stiffness. This relation holds due to plastic incompressibility, i.e., $\det\;F^{p}=1$.

At this stage, a constitutive model must be incorporated to acquire $F^{p}$ and further kinetics representations. The next section describes the rationale behind the developed constitutive model.

### 2.2. Entropic Kinetics and Constitutive Model at Nano-Scale

Considering metals and metallic alloys with nanoscale size under generic loading conditions, stress- and temperature-driven interface and surface evolution phenomena bear significance in mechanical responses. Dislocation-mediated deformations are entropy sources compelling a system towards a higher probability state independent of ordinary thermal fluctuations. Thus, a system kinematics must encompass dislocation-specific thermodynamics representation [27,28]. Assuming quasi-static transitions in all configurations, the contributions due to total dynamic quantities are negligible, however, the system is not necessarily in thermodynamic equilibrium. Total entropy generation per unit time, Γ(t), as the difference between the reference rate of change of entropy, $\dot{S}=\frac{DS}{Dt}$, and the rate of entropy input, $\dot{Q}$, of a body with volume Λ and boundary surface ∂Λ follows the global format in the reference configuration with

(1) $$\begin{array}{ccc} \Gamma (t) & = & \frac{DS}{Dt}-\dot{Q}=\frac{D}{Dt}\int_{\Lambda }\eta \left(\lambda ,t\right)\;dv+\int_{\partial \Lambda }H\left(\lambda ,t\right)\cdot n\;ds-\int_{\Lambda }R\left(\lambda ,t\right)\;dv\geq 0\;\\ \; & = & \frac{D}{Dt}\int_{\Lambda }\eta \left(\lambda ,t\right)\;dv+\int_{\partial \Lambda }\frac{Q(\lambda ,t)}{\Theta (\lambda ,t)}\cdot n\;ds-\int_{\Lambda }\frac{R(\lambda ,t)}{\Theta (\lambda ,t)}\;dv\geq 0\;, \end{array}$$

where η(λ,t) is the entropy per unit volume as a function of material position vector, λ, and time, *t*, H(λ,t) the true entropy flux, Q(λ,t) the material heat flux, n the normal boundary surface vector, Θ(λ,t) the thermal scalar field, and R(λ,t) the entropy source. Since the rate of entropy change is always greater than the rate of entropy input, the total entropy generation is time- and direction-dependent specifying the irreversibility of thermodynamical processes [29,30] including plastic deformation. The rate of thermal work involving the total heat flux and source is inversely related to the rate of entropy input through the thermal scalar field and first Piola–Kirchhoff stress, P(λ,t). Based on the divergence theorem, $\int_{s}\Psi v\cdot n\;ds=\int_{v}\nabla \cdot \left(\Psi v\right)dv$, the local form of the entropy inequality can be expressed as

(2) $$\dot{\eta }\left(\lambda ,t\right)-\frac{R(\lambda ,t)}{\Theta (\lambda ,t)}+\frac{1}{\Theta (\lambda ,t)}\nabla \cdot Q\left(\lambda ,t\right)-\frac{1}{\Theta^{2}\left(\lambda ,t\right)}Q\left(\lambda ,t\right)\cdot \nabla \Theta \left(\lambda ,t\right)\geq 0\;,$$

and

(3) $$\Theta \left(\lambda ,t\right)\;\dot{\eta }\left(\lambda ,t\right)\;+\frac{1}{\Theta (\lambda ,t)}Q\left(\lambda ,t\right)\cdot \nabla \Theta \left(\lambda ,t\right)+P\left(\lambda ,t\right):\dot{F}-\dot{e}\geq 0\;,$$

in which the local form of balance of energy, $\dot{e}=P\left(\lambda ,t\right):\dot{F}-\nabla \cdot Q\left(\lambda ,t\right)+R\left(\lambda ,t\right)$, is considered, where ∇ is the differential operator. The heat conduction inequality, Q(λ,t)·∇Θ(λ,t)≤0, applies a constraint on the heat flux vector, i.e., there is no heat flux without a temperature. For elastoplastic behavior of a crystal, the local entropy production, $\Upsilon_{in}$, is positive or at least zero where heat flux approaches to zero in reversible processes, i.e.,

(4) $$\Upsilon_{in}=P\left(\lambda ,t\right):\dot{F}-\dot{e}+\Theta (\lambda ,t)\;\dot{\eta }\left(\lambda ,t\right)\geq 0\;.$$

Therefore, the internal dissipation stems from three sources; the rate of internal mechanical work per unit volume, the rate of internal energy, and the absolute temperature coupled with the rate of entropy, respectively. With the aid of the balance of energy, the lower bound of Equation (4) can be represented by

(5) $$\Theta (\lambda ,t)\;\dot{\eta }\left(\lambda ,t\right)=\Upsilon_{in}+R\left(\lambda ,t\right)-\nabla \cdot Q\;.$$

The constitutive relation Q(λ,t)=ϰ∇Θ(λ,t) relates the the material heat flux to the temperature gradient with thermal conductivity, ϰ, as a function of deformation and temperature. The condition of $\varkappa \nabla^{2}\Theta \left(\lambda ,t\right)\geq 0$ signifies the elliptical nature of the constitutive equation for which the solutions are accordingly to be realized. For an adiabatic thermodynamic process, $P_{\left(a\right)}$, the thermal power and the rate of entropy input are zero since thermal energy can not traverse the system boundary surfaces, be generated, or destroyed. In this circumstance, no decay in the total entropy is possible while that is not the case for point-wise entropy that degenerates the energy balance equation and annihilates that for an additional reversibility condition, $P_{\left(a,r\right)}$ [31].

(6) $$\left\{\begin{array}{cc} \Theta \left(\lambda ,t\right)\dot{\eta }=\Upsilon_{in} & \;\;;\;\;P_{\left(a\right)}\\ \Theta \left(\lambda ,t\right)\dot{\eta }=0 & \;\;;\;\;P_{\left(a,r\right)} \end{array}\right.$$

Considering the anisotropic elastoplastic behavior of crystalline materials, internal variables need to be independently assimilated in any thermodynamical approaches, hence, the Helmholtz free-energy function, $H(F,\Theta \left(\lambda ,t\right),V_{i})$, is defined as a function of deformation gradient, thermal field, and internal variables, $V_{i}\;;\;i=1,...,n$, respectively. Here, $V_{i}$ represents plastic deformation mechanisms, associated with any admissible inelastic phenomena, *n*, for presumed external thermomechanical loading conditions. Thus, the Helmholtz free-energy function can be described in terms of the internal dissipation inequality as

(7) $$\Upsilon_{in}=P\left(\lambda ,t\right):\dot{F}-\dot{H}\left(F,\Theta \left(\lambda ,t\right),V_{i}\right)-\dot{\Theta }\left(\lambda ,t\right)\;\eta \left(\lambda ,t\right),V_{i})\geq 0\;,$$

where the chain rule time differentiation of the Helmholtz free-energy function yields

(8) $$\begin{array}{ccc} \dot{H}\left(F,\Theta \left(\lambda ,t\right),V_{i}\right) & = & {\left(\frac{\partial H(F,\Theta \left(\lambda ,t\right),V_{i})}{\partial F}\right)}_{\Theta ,V_{i}}:F+{\left(\frac{\partial H(F,\Theta \left(\lambda ,t\right),V_{i})}{\partial \Theta }\right)}_{F,V_{i}}\dot{\Theta }\left(\lambda ,t\right)\\ \; & + & \sum_{i=1}^{n}{\left(\frac{\partial H(F,\Theta \left(\lambda ,t\right),V_{i})}{\partial V_{i}}\right)}_{F,\Theta }:\dot{V_{i}}\;\; \end{array}$$

The acquired constitutive equation involves stress, thermal flux, and internal entropy associated with plastic deformations through internal strain rates, ${\dot{V}}_{i}$, for which

(9) $$\Upsilon_{in}=\sum_{i=1}^{n}D_{i}:\dot{V_{i}}\geq 0\;\;\;;\;\;\;D_{i}=-{\left(\frac{\partial H_{i}\left(F,\Theta \left(\lambda ,t\right),V_{i}\right)}{\partial V_{i}}\right)}_{F,\Theta }\;,$$

is deduced in terms of internal dissipations, $D_{i}$. For the stretch type deformation, compatible with dislocation mechanisms, considering symmetric internal variables, $U_{i}$, and stretch tensor, C, Equation (9) becomes

(10) $$\sum_{i=1}^{n}\frac{\partial H_{i}\left(C,\Theta \left(\lambda ,t\right),U_{i}\right)}{\partial U_{i}}:\dot{U_{i}}\leq 0\;,$$

where the rate of the symmetric internal variables, ${\dot{U}}_{i}$, evolves in irreversible mechanisms with a designated function, E, through ${\dot{D}}_{i}=E\left(C,\Theta \left(\lambda ,t\right),U_{1},U_{2},...,U_{n}\right)$. The second Piola–Kirchhoff stress, S, is involved in the evolution equations due to its dependence on the free energies of either reversible or irreversible processes which represents that as a function of external variables, S=S(C,Θ(λ,t)).

The internal states of disorder in a crystalline solid contain the majority of barriers including point defects, grain boundaries, junctions, intersections, locks, stacking faults, or combinations of those in dislocation dynamic annihilations and recoveries [28].

The average velocity of dislocations, $v_{d}$, and the time spent between obstacles, $t_{d}$, both are functions of applied stress, τ, and absolute temperature, *T*, which define the dislocation mean free path with $l_{d}=t_{d}\left(\tau ,T\right)\;v_{d}\left(\tau ,T\right)$. The probability function indicating the possible states of slip due to thermal fluctuation and applied loading is $P_{s}=\exp\left(-\frac{\Delta G}{k_{B}T}\right)$, where ΔG is the activation free enthalpy and $k_{B}$ the Boltzmann constant. If a dislocation is effectively vibrating with the frequency of $f_{d}$, it successfully overcomes barriers at a rate of ${\dot{P}}_{s}=f_{d}\exp\left(-\frac{\Delta G}{k_{B}T}\right)$, therefore, the dislocation velocity associated with the mean free path for each conquered obstacle with the presence of thermal fluctuation will be $v_{d}=l_{d}f_{d}\left[\exp\left(-\frac{\Delta G}{k_{B}T}\right)\right]$.

The dependence of flow strength on temperature and applied stress is determined based on the required energy to overcome any types of barriers while dislocations slip. In a general nonlinear temperature-dependent case it can be demonstrated in terms of the Helmholtz free energy as

(11) $$\Delta G=\Delta H{\left[1-{\left(\frac{\left|\tau^{\alpha }\right|-\tau_{r}^{\|}}{\tau_{r}^{⊥}}\right)}^{p}\right]}^{q}\;,$$

where $\tau^{\alpha }$ is the resolved shear stress and $\tau_{r}^{\|}$ and $\tau_{r}^{⊥}$ are the resistance stresses parallel and orthogonal to slip surface, respectively. The exponents, *p* and *q*, can be initially identified with mechanistic assessments and finalized through calibration processes.

The rate of Helmholtz energy in Equation (8) is a function of external and internal dissipative variables. Consequently, the energy needed to overcome hindrances is manifested in deformation mechanisms controlled by thermal activation and applied loading [32,33]. Considering the alternating directions of dislocation motion over an obstacle, thermally activated plastic shear strain rate for each slip system can be expressed as $\dot{\gamma }=\rho_{m}^{\alpha }\;b\;l_{d}^{\alpha }\;f_{d}^{\alpha }\sinh\left(-\frac{V_{a}}{k_{B}T}\left(\left|\tau^{\alpha }\right|-\tau_{r}^{\|}\right)\right)\exp\left(-\frac{\Delta H}{k_{B}T}\right)$, where $V_{a}$ is the activation volume. The pre-exponential terms can be merged into the initial plastic strain rate, ${\dot{\gamma }}_{0}^{\alpha }=\rho_{m}^{\alpha }bl_{d}^{\alpha }f_{d}^{\alpha }=\rho_{m}^{\alpha }b^{2}f_{D}$, where $f_{D}$ denotes the Debye frequency of constituents. This term is considered a slow variable since the change of mobile dislocation density is not decisive relative to that of free energy, yet, not completely uneventful.

The obstacles surmounted by thermal energy and mechanical work are described by $\left(\left|\tau^{\alpha }\right|-\tau_{r}^{\|}\right)\;V_{a}$ [27]. In metals with nano-range sizes, activation volumes decline significantly due to diminished local volumes involved in the depinning of a propagating dislocation [34], thermally activated mechanism generating interface mediated dislocations [35], and so forth. Thereby, considering an increase in a system entropy due to thermal fluctuations, internal and external state variables, statistical probabilities of dislocation positions, and unidirectional dislocation jumps, a constitutive model is developed at the size spectrum of 20 nm to 1 μm encapsulating size and constituent effects in the abrupt variations of activation volume, stress concentration, and complex dislocation mechanisms with

(12) $${\dot{\gamma }}^{\alpha }=\left\{\begin{array}{cc} {\dot{\gamma }}_{0}^{\alpha }\exp\left\{-\frac{Q_{active}}{k_{B}T}{\left[1-{\left(\frac{{\left(\tau_{\mathrm{eff}}^{\alpha }\right)}^{2}}{\tau_{\mathrm{cut}}^{\alpha }}\frac{c_{s}\pi }{\mu b}\;d\right)}^{p}\right]}^{q}\right\}\mathrm{sgn}\left(\tau^{\alpha }\right) & \tau_{\mathrm{eff}}^{\alpha }>0\\ 0 & \tau_{\mathrm{eff}}^{\alpha }\leq 0 \end{array}\right.$$

The model is constructed based on exclusively addressing size effects in *d* as the layer thickness and the constituent type and morphology through $c_{s}$ as the material shape parameter. The effective shear stress is defined as $\tau_{\mathrm{eff}}^{\alpha }=\left|\tau^{\alpha }\right|-\tau_{\mathrm{pass}}^{\alpha }$, where passing, $\tau_{\mathrm{pass}}^{\alpha }$, and cutting, $\tau_{\mathrm{cut}}^{\alpha }$, stresses are athermal and thermal shear resistances, respectively, $Q_{active}$ is the activation energy, and μ the shear modulus. Long-range athermal resistances stem from the composition, heat treatment, and dislocation structure of the material including the stress fields of other dislocations and incoherent inclusions, while short-range thermal barriers involve sources such as the Peierls-Nabarro force, stress fields of coherent inclusions, cross slip, climb, and dislocations intersections.

Plasticity initiation is recognized when the effective shear stress is positive while elastic behavior is resumed otherwise. The athermal resistance is designed with an evolution in terms of contrasting slip systems, β, by

(13) $${\dot{\tau }}_{pass}^{\alpha }=\sum_{\beta =1}^{N}h^{\alpha \beta }\left|{\dot{\gamma }}^{\beta }\right|\;,$$

where ${\dot{\gamma }}^{\beta }$ specifies the effect of other N slip systems considering the initial value of the athermal resistance, $\tau_{\mathrm{pass}-\mathrm{init}}^{\alpha }$, and $h^{\alpha \beta }$ includes both self and latent hardening with

(14) $$h^{\alpha \beta }=\left\{\begin{array}{cc} \sum_{\beta =1}^{N}h_{0}^{\beta }{\left|1-\frac{{\left|\left|\tau_{cut}^{\beta }+\tau_{pass}^{\beta }\right|\right|}_{2}}{\tau_{sat}^{\beta }}\right|}^{r}\left[q^{\alpha \beta }+\left(1-q^{\alpha \beta }\right)\delta^{\alpha \beta }\right] & {\left|\left|\tau_{cut}^{\beta }+\tau_{pass}^{\beta }\right|\right|}_{2}\leq \tau_{sat}^{\beta }\\ 0 & {\left|\left|\tau_{cut}^{\beta }+\tau_{pass}^{\beta }\right|\right|}_{2}>\tau_{sat}^{\beta } \end{array}\right.$$

in which ${\left||\;x\;|\right|}_{2}$ denotes the Euclidean norm, $h_{0}^{\beta }$ is the initial hardening, $q^{\alpha \beta }$ a magnitude for self and latent hardening considered 1.0 for coplanar slip systems and 1.4 otherwise, *r* the hardening exponent, and $\delta^{\alpha \beta }$ the slip systems Kronecker delta function. $\tau_{sat}^{\beta }$ is the saturation shear stress designed as $\tau_{sat}=c_{sat}\;d^{m}$, where $c_{sat}$ and *m* are the saturation coefficient and exponent, respectively.

In order to utilize the constitutive model in crystal plasticity procedures and solve system equations, derivation techniques and computational approaches are required to be carried out as delineated in Appendices Appendix A and Appendix B. Inevitably, multiple parameters in the model must be realized, as demonstrated in Table 1, for which the next section is assigned.

### 2.3. Deep-Learning Single Layer Calibration (SLC) Method

In order to acquire constitutive parameters a deep-learning single-layer calibration (SLC) method is developed. This technique is able to reduce the experimental data, recognize and distinguish dominant and trivial patterns, and efficiently decide trade-offs between bias and variance paths along with some other optimization, recognition, and decision capabilities.

#### Training and Learning Techniques in The SLC Approach

This approach is based on adaptive boosting technique [36] over a committee of models. The operation proceeds through combining classifiers, $M_{j}\;;\;j=1,2,...,m$, by sequentially training *n* models and concluding the final prediction based on the ultimate outcome. One of the main advantages of this procedure is obtaining favorable results even if the base classifiers are not strong learners. However, the possibility of achieving solid outcomes exponentially increases in the cases of moderate or advanced classifiers that are believed characterizes the current work features. Boosting can be extended to regression problems [37] which in some optimization stages is employed as well.

Here, the classifiers are trained using a weighted array in which the coefficient values depend on the performances of the previous classifiers. The descending sorted coefficients are proportional to the level of the misclassification of data and are key in the final decision. At the beginning, each of *n* weighting coefficient, $c_{w}^{\left(i\right)}$, in the array is uniformly initialized based on the data point vectors, $v_{i}$, and binary target values, $t_{i}\in \left\{-1,1\right\}\;\;,\;\;i=1,2,..,n$, among the classifiers as $c_{w}^{\left(i)(j\right)}=\frac{1}{n}$. Then, each model is trained while the associated weighted error function, $e_{j}=\sum_{i=1}^{n}c_{w}^{\left(i)(j\right)}N\left(M_{j}\left(v_{i}\right)\neq t_{n}\right)$, is minimized, where $N(M_{j}\left(v_{i}\right)\neq t_{n})$ is the indicator function. Weighting coefficients are continuously adjusted for succeeding models by a modifier,

(15) $$\psi_{j}=\left(\frac{1-\sum_{i=1}^{n}c_{w}^{\left(i)(j\right)}N\left(M_{j}\left(v_{i}\right)\neq t_{n}\right)}{\sum_{i=1}^{n}c_{w}^{\left(i)(j\right)}}\right)\;,$$

allocating larger weighting values to more precise classifiers. Afterwards, the weighting coefficient array is updated by

(16) $$c_{w}^{\left(i+1)(j\right)}=c_{w}^{\left(i)(j\right)}{\left(\frac{\sum_{i=1}^{n}c_{w}^{\left(i)(j\right)}}{\sum_{i=1}^{n}c_{w}^{\left(i)(j\right)}N\left(M_{j}\left(v_{i}\right)\neq t_{n}\right)}-1\right)}^{N(M_{j}\left(v_{i}\right)\neq t_{n})}\;.$$

The exponential error function [38] is defined as $E=\frac{1}{2}\sum_{i=1}^{n}\sum_{j=1}^{m}\exp\left[-t_{i}\psi_{j}M_{j}\left(v_{i}\right)\right]$ which is sequentially minimized in terms of $\psi_{j}$ and $M_{j}\left(v_{i}\right)$. This relation holds with the assumptions of fixed base classifiers and their modifiers.

**Figure 1:**
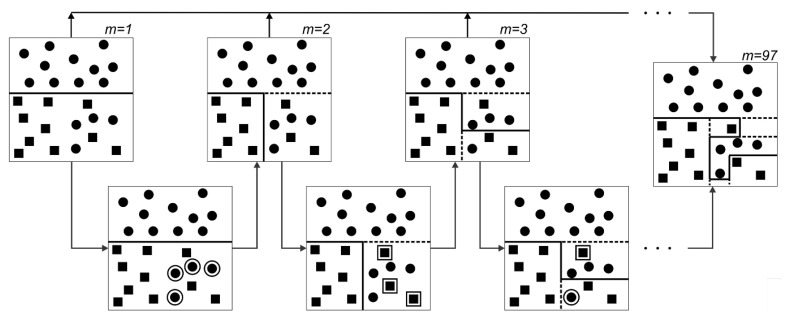
Demonstration of adaptive boosting technique where base classifiers with simple thresholds are trained according to the assigned weighted function acquired relative to the precision of the previous classifier in data allocation. Each sample shows the number of classifiers, *m*, trained up to that point. The solid and dashed lines in the domains are the decision made and revised choices, respectively, based on the weight of the misplaced data illustrated with expanded boundaries.

The boosting framework is illustrated schematically in Figure 1 where each base classifier is trained according to the assigned weighted function acquired in terms of the precision of previous classifiers in data allocation with the error function between two consecutive classifiers as

(17) $$E=\sum_{i=1}^{n}\sum_{j=1}^{m}\exp\left(-\frac{1}{2}\left[t_{i}\psi_{j-1}M_{j-1}\left(v_{i}\right)-t_{i}\psi_{j}M_{j}\left(v_{i}\right)\right]\right).$$

If data points are divided into proper, $C_{j}^{+}$, and improper, $C_{j}^{-}$, categories, Equation (17) can be restated by

(18) $$\begin{array}{ccc} E & = & \sqrt{\psi_{j}-1}\sum_{i\in C_{j}^{+}}c_{w}^{\left(i)(j\right)}+\sqrt{\frac{1}{\psi_{j}}-1}\sum_{i\in C_{j}^{-}}c_{w}^{\left(i)(j\right)}\\ \; & = & \left(\sqrt{\psi_{j}-1}-\sqrt{\frac{1}{\psi_{j}}-1}\;\right)\sum_{i=1}^{n}c_{w}^{\left(i)(j\right)}N\left(M_{j}\left(v_{i}\right)\neq t_{n}\right)+\sqrt{\frac{1}{\psi_{j}}-1}\sum_{i=1}^{n}c_{w}^{\left(i)(j\right)}\;. \end{array}$$

Thus, from Equations (17) and (18) and $t_{i}M_{j}\left(v_{i}\right)=1-2N\left(M_{j}\left(v_{i}\right)\neq t_{n}\right)$, weighting coefficients are modified as

(19) $$c_{w}^{\left(i+1)(j\right)}=c_{w}^{\left(i)(j\right)}\exp\left(-\frac{1}{2}t_{i}\psi_{j}M_{j}\left(v_{i}\right)\right)=c_{w}^{\left(i)(j\right)}\sqrt{\psi_{j}-1}{\left(\frac{1}{\psi_{j}}-1\right)}^{\frac{N(M_{j}\left(v_{i}\right)\neq t_{n})}{2}}\;.$$

Finally, when the training of the classifiers are completed, the sign of the combined function for each data point vector is obtained with

(20) $$\mathrm{SGN}\left(v_{i}\right)=\mathrm{sgn}\left[M_{j}\left(v_{i}\right)\;\ln\left(\frac{\sum_{j=1}^{m}c_{w}^{\left(i)(j\right)}}{\sum_{i=1}^{n}c_{w}^{\left(i)(j\right)}N\left(M_{j}\left(v_{i}\right)\neq t_{n}\right)}-1\right)\right].$$

### 2.4. Statistical Mechanics and Homogenized Crystal Plasticity Constitutive Model

The notion of multi-scale modeling has been of constant interest in the realm of computational mechanics and materials. Despite diverse length-scale-dependent methods, hierarchical systems are able to resolve the geometrical and physical details of the underlying mechanisms in lower-scale with higher speed of computation, yet, reasonable precision and simplicity. The reliability extent of acquired responses is evaluated by the adequacy of lower scales assessments, that is, these levels exhibit momentous complementary effects.

Classical thermodynamics relations need to be revised for homogenized solids due to the fundamental differences in the degrees of freedom with gases and fluids especially when the goal is eliminating fast atomic degrees of freedom and attaining a homogenization theory. In the case of crystals, elimination is related to the dynamics of crystal defects, particularly dislocations, leading to an additional coarse-graining with the system of governing equations that is no longer Hamiltonian, but dissipative. Unlike ergodic systems characterized by macrovariables and energy parameters, dissipative systems are extremely diverse [39] for which developing frameworks is possible with specific considerations.

Here, the statistical mechanics of the microcanonical ensemble [40] are utilized for the lower scale since the upper scale kinematics are to be characterized considering dissipative transport and nonlinear geometrical models of dislocations [41] along with the independent point-wise temperature. The Clausius-Duhem inequality is incorporated to link the scales through entropy flux where the probability of a phase-space invariant measure with probability density function is assumed plausible.

Dislocation positions are not statistically independent and affect the overall energy of a nano-structure, however, since the precise instantaneous locations associated with the initially considered dislocations can not be identified as deformation evolves, energy is considered as an independent additional characteristic of a dislocation geometrical network [42]. Thereby, the total number of the dislocations, $N_{d}$, and associated energy, $E_{d}$, are the independent features of the dislocation network with the priori of equal probability of the ensemble sub-states.

If x denotes the position of a material point in a system at time *t* with the continuum mass density, ρ(x,t), divided into $N_{ns}$, total sub-nano-systems, with identical masses, $m_{ns}$, and individual volumes, $V_{ns}^{i}$, in the total spatial volume, Λ, the system average velocity is defined as $\langle v\rangle =\frac{1}{N_{ns}}\sum_{i=1}^{N_{ns}}v_{ns}^{i}$. Velocity fluctuation is determined through ${\tilde{v}}_{ns}^{i}=\langle v\rangle -v_{ns}^{i}$, where $v_{ns}^{i}$ is the velocity of each sub-system. The dissipative nature of a system is characterized via the velocity fluctuation of each sub-system, hence, the system disorder manifests itself in the total energy of a system from the lower-scale standpoint as

(21) $$\frac{m_{ns}}{2}\left(\langle v\rangle \cdot \langle v\rangle +\sum_{i=1}^{N_{ns}}{\tilde{v}}_{ns}^{i}\cdot {\tilde{v}}_{ns}^{i}\right)+U{|}_{U_{ns}^{i}\;;\;\varepsilon^{i}}\;,$$

where *U* is the total potential energy acquired from the subsystems. The internal energy, $U_{ns}^{i}$, is determined over a surrounding volume, $V_{ns}^{i}$, such that the deviation at each infinitesimal part of the volume surface, $\varepsilon^{i}$, depends on the long- and short-range interactions considered among dislocations therein, thus, varies by time evolution, heat flux, and active deformation mechanisms. This relation is associated with the total energy of an ensemble stated by $\int_{\Lambda }\rho \left(x,t\right)\left(\frac{1}{2}\overset{ˇ}{v}\cdot \overset{ˇ}{v}+e\right)dv$, with $\overset{ˇ}{v}$ as the velocity and e as the internal energy density of the system. Consequently, the Helmholtz free energy,

(22) $$H=U_{s}{|}_{U_{ns}^{i}\;;\;\varepsilon^{i}}+\frac{m_{ns}}{2}\sum_{i=1}^{N_{ns}}{\tilde{v}}_{ns}^{i}\cdot {\tilde{v}}_{ns}^{i}=U-TS\;,$$

is obtained in which the entropy, S, is associated with the subsystems velocity fluctuations.

**Figure 2:**
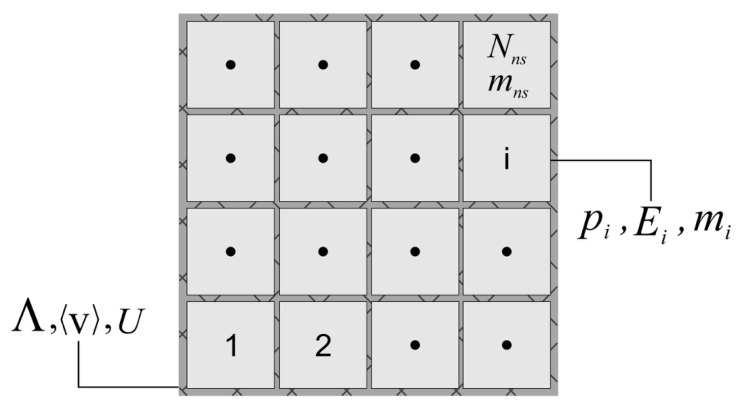
Schematic representation of a microcanonical ensemble with equal probability of state, $p_{i}$, and energy, $E_{i}$, of each subsystem in the total volume, Λ, with the average velocity of 〈v〉 and energy *U*.

In order to link the lower and upper scale, the statistical Boltzmann entropy principle is utilized as

(23) $$S=-k_{B}\sum_{i}p_{i}\ln p_{i}$$

(24) $$p_{i}=\frac{\exp(-\frac{E_{i}}{k_{B}T})}{\sum_{i}\exp\left(-\frac{E_{i}}{k_{B}T}\right)}\;,$$

where $p_{i}$ is the probability of the *i*th subsystem with $E_{i}$ energy as displayed in Figure 2. The hypothesis of an ensemble with a uniform probability distribution in phase-space, necessitates the confinement of a subsystem in a particular volume with constant total energy, thus, the system entropy has the format of

(25) $$\begin{array}{ccc} S & = & -k_{B}\sum_{i}\frac{\exp(-\frac{E_{i}}{k_{B}T})}{\sum_{i}\exp\left(-\frac{E_{i}}{k_{B}T}\right)}\ln\left(\frac{\exp(-\frac{E_{i}}{k_{B}T})}{\sum_{i}\exp\left(-\frac{E_{i}}{k_{B}T}\right)}\right)\\ \; & = & -k_{B}\sum_{i}\frac{\exp(-\frac{E_{i}}{k_{B}T})}{\sum_{i}\exp\left(-\frac{E_{i}}{k_{B}T}\right)}\left(-\frac{E_{i}}{k_{B}T}\right)+k_{B}\sum_{i}\frac{\exp(-\frac{E_{i}}{k_{B}T})}{\sum_{i}\exp\left(-\frac{E_{i}}{k_{B}T}\right)}\ln\sum_{i}\exp\left(-\frac{E_{i}}{k_{B}T}\right). \end{array}$$

Comparing Equation (25) with Equation (22) results

(26) $$U=\sum_{i}E_{i}\;p_{i}=\langle E\rangle \;,$$

where 〈E〉 corresponds to the average energy of the subsystems. Here, boundary conditions of the homogenized medium presume no relative fluctuations, thus, the extensive variables in the upper scale follow the average principles whose the plausibility is proven.

The homogenized crystal plasticity-based model is founded upon the continuum slip theory of generalized Taylor scale-transition [43,44]. It contains parameterized representation of the nano-structure features with embedded rate-dependence and latent hardening effects accounting for thermomechanical properties in both elastic and plastic responses. The concept of the representative volume element (RVE) statistically representing the nano-system is incorporated based on retaining the relative dimensions between the homogenized ensemble and nano-structures as well as the underlying deformation mechanisms and dominant features. The hierarchical homogenization analysis follows the Hill–Mandel principle of macro-homogeneity [45,46] where the volume average of the work increment applied on an RVE is considered equal to the variation of the work on the homogenized system. In the absence of body forces and inertia, the energy consistency is stated in terms of the Eulerian strain rate, $\dot{e}$, and Cauchy stress with

(27) $$\frac{1}{V_{n}}\int_{\Lambda }\sigma_{n}:{\dot{e}}_{n}\;dV_{n}=\sigma_{H}:{\dot{e}}_{H}\;,$$

where $V_{n}$ is the volume of the RVE and subscripts *n* and *H* correspond to nano- and homogenized systems, respectively. Considering the quasi-static applied strain rates, the self-equilibrated spatial stress field is achieved by ∇·σ=0. In order to solve the boundary value problem in Equation (27) and equilibrium equations, a homogenized crystal plasticity-based constitutive model with the identical nano-scale model backbone, yet, a simplified structure is developed as

(28) $${\dot{\gamma }}^{\alpha }={\dot{\gamma }}_{0}^{\alpha }\exp\left[-\frac{Q_{active}}{k_{B}T}\left(1-\frac{\tau_{\mathrm{eff}}^{\alpha }}{\tau_{\mathrm{cut}}^{\alpha }}\right)\right]\mathrm{sgn}\left(\tau^{\alpha }\right)\;.$$

Considering previously defined parameters, the CPFE approach is utilized to solve the equilibrium equation as described in appendices A and B. The constitutive parameters are formulated in terms of structural variables and calibrated through the computational homogenization of the lower scale model and the RVE that consists of layer thicknesses of stacked nano-layers.

Prior to plasticity, the elastic responses of a homogenized system must be realized, thus, the equivalent elastic constants, ${\overset{ˇ}{C}}_{ij}$, are attained as a combination of the constituents elastic constants, $C_{ij}$, with respect to their thicknesses, $d_{k}$, in a multi-nano-layer as ${\overset{ˇ}{C}}_{ij}=\sum_{k=0}^{N_{mat}}C_{ij}\frac{d_{k}}{d}$, where *d* is the total thickness of the specimen and $N_{mat}$ the number of materials. The rate dependence feature is modified for the homogenized ensemble with the total $N_{l}$ layers by $l^{p}=\sum_{i=1}^{N_{l}}\lambda^{i}\;{\dot{\gamma }}^{i}\;\left(m_{0}^{i}⊗n_{0}^{i}\right)$ in which $\lambda^{i}=\frac{V_{i}}{V_{total}}$ signifies each layer volume fraction.

## 3. Results and Discussion

In this section, the results are categorized into two nano- and homogenized scale where Cu/Nb multi-layers are designated as case studies for both regimes.

At the nano-scale, the deep-learning SLC method is utilized to calibrate and validate material parameters by experimental data where the constitutive model predictive capabilities are demonstrated. Subsequently, the nano-layer responses are predicted and discussed regarding size and constituent effects, the extent of impacts in variation of layer and/or loading orientation, and the influence of layer setups in the initial conditions of calibration settings.

At the homogenized level, deep-learning SLC and genetic algorithms are utilized to realize and obtain effective functionals relations and constants, then, the results are favorably compared with the nano-scale model while expediting the computational processes by several orders of magnitude. Further assessments of temperatures effects on the nano-metals properties are performed for which deep-learning SLC and genetic algorithms are utilized to realize and obtain effective functionals relations and constants utilizing multiple experimental results which also incorporated for final validations. Ultimately, several responses regarding the effects of elevated temperature and the degradation of properties are predicted.

### 3.1. Nano-Scale Constitutive Parameters and Predictions

The presented deep-learning SLC approach utilizes the single crystal stress-strain curve of each constituent and delivers generalized parameters via a single process applicable to a broad scope of setups that are entirely different than those of the calibration ones. The models in the committee are defined based on the developed constitutive model and variation of each parameter considered as an independent variable along with the cases that assume simultaneous parameter variation effects. The developed SLC method is an identifier of constitutive and effective parameters based on the physics behind the role of the parameters on the overall behavior of the concerned material. Thereby, it trains and realizes the best compatible parameters in the constitutive model while applicable to a broad range of material morphologies.

Here, several Cu and Nb nano-layers are separately simulated for which elastic constants are Initially obtained through analytical processes and databases displayed in Table 2.

Subsequently, the constitutive parameters are obtained, Table 3, via experimental data of single crystalline Cu [47] and Nb [48]. The exponential error functions, E, associated with training segments are defined based on the binary target values and classifiers while minimized iteratively through modifiers, where the range of variation is captured from 0.008% to 0.01% for which static thresholds of ≤0.05% are designated.

**Figure 3:**
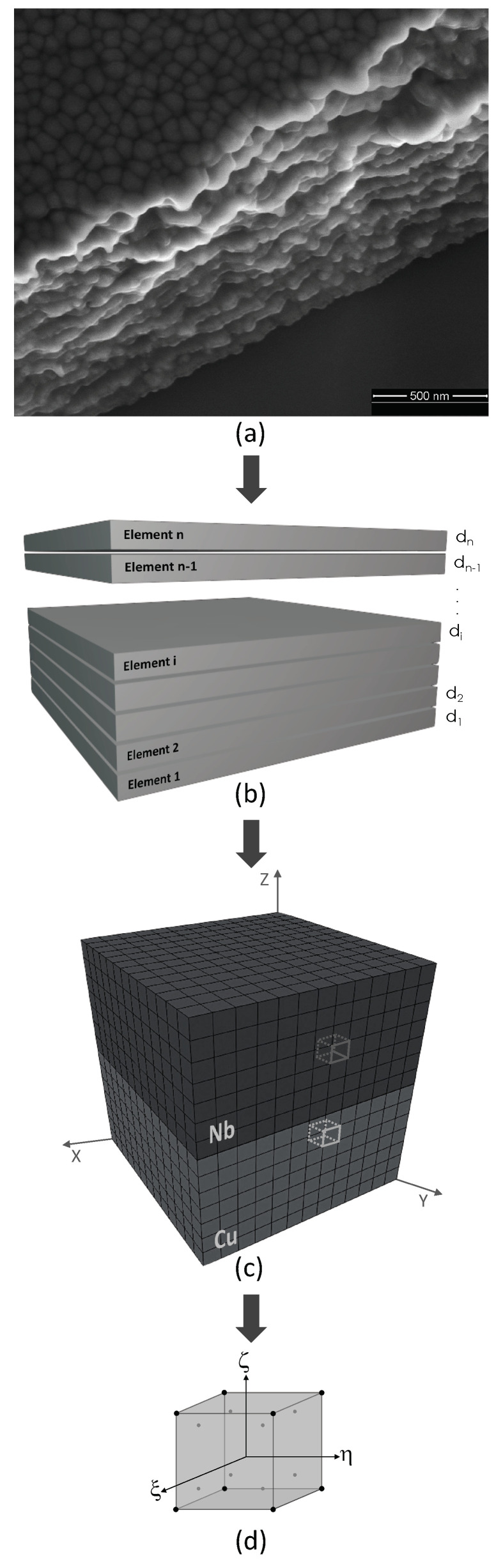
(**a**) A high resolution scanning electron microscopy image of a metallic nano-layer. (**b**) A generalized representative structure of a metallic nano-layer with *n* elements/layers. (**c**) A 3-dimensional Cu/Nb nano-layer unit cell discretized into (**d**) eight-node hexahedral elements with eight integration points and the local coordinate system of (ξ,η,ζ).

Sequential modeling steps from an actual metallic nano-layer image to a three-dimensional Cu/Nb nano-layer unit cell discretized into hexahedral elements are demonstrated in Figure 3.

**Figure 4:**
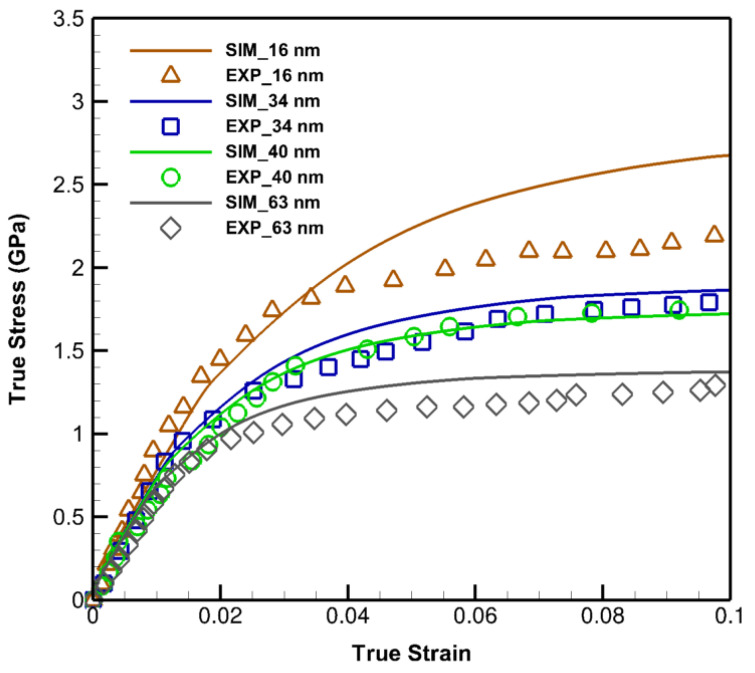
The verification of the size-dependent constitutive model and deep-learning SLC results plotted by “SIM” and solid lines with the experimental data [49,50] designated by “EXP” and symbolic points.

In order to illustrate the capabilities of the developed models in generic perspectives and demonstrate the accuracy of the SLC method, additional simulations are performed utilizing the acquired parameters. The responses of the specimens in the form of true stress-strain curves are compared with the experimental results in [49,50], Figure 4, having entirely different setups than those of the calibrations in [47,48]. The engineering stress-strain curves in [49] are obtained for the average layer thicknesses of 16 nm, 34 nm, and 63 nm under the constant strain rate of $10^{-3}$/s while a true stress-strain curve is achieved in [50] involving the average layer thickness of 40 nm with $2\times 10^{-4}$/s strain rate. The experimental and simulations are performed with the Kurdjumov–Sachs (KS) orientation relationships, {111}Cu||{110}Nb.

Since the developed models yield true stress-strain responses, an excellent agreement with 40 nm experimental data is observed due to the similarity of formats. Small divergences between the rest of the curves are related to the nature of the reported results, being engineering stress-strain, which naturally placed them in the lower positions than the true ones. The general trend of the computationally predicted properties is in agreement with the experimental data, however, the amount of deviation from 16 nm is related to the softening phenomenon at the sizes lower than about 20 nm [51,52] due to which this work is appointed its nano-scale size range from 20 nm to 1 μm.

At this stage, considering the models that are validated and also the validities are solidly tested, several predictive case studies are assessed and discussed.

**Figure 5:**
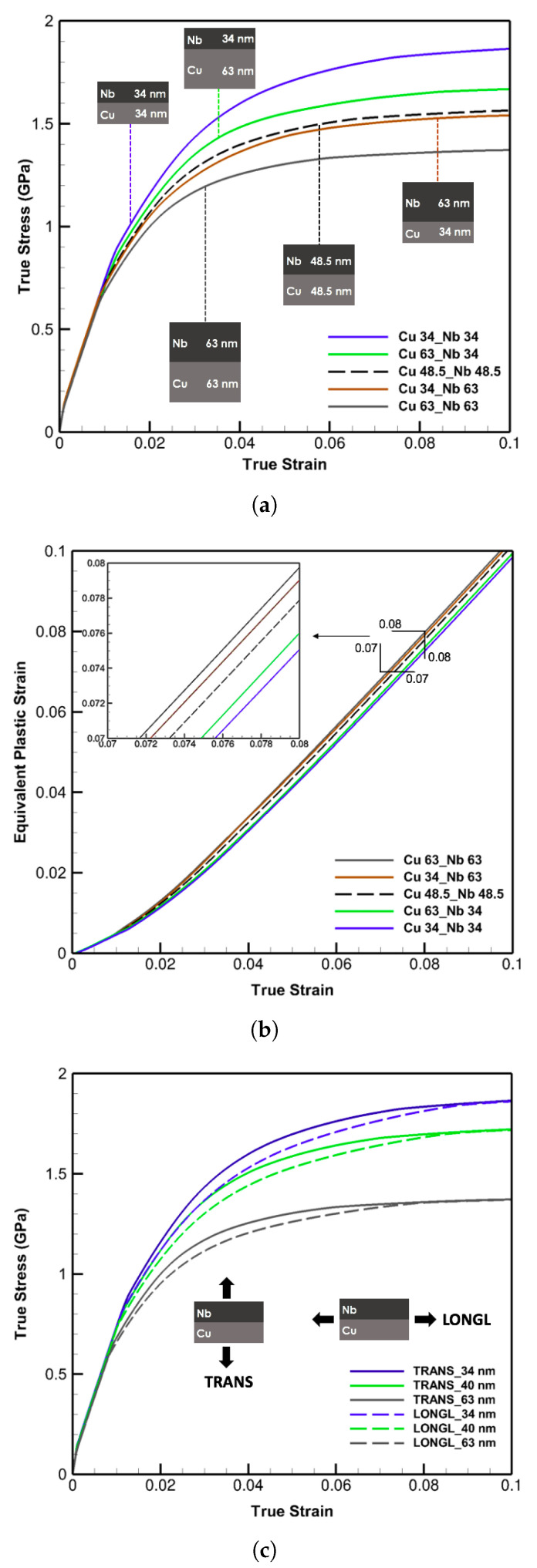
(**a**) True stress-strain curves for four thickness combinations of 34 nm and 63 nm as well as 48.5 nm Cu/Nb multi-layers illustrating the effect of layer combinations on the plastic deformation and flow strength. (**b**) Equivalent plastic strain versus true strain curves for the cases in (**a**) clarifying the size and layer geometrical order effects. (**c**) True stress-strain curves of 34 nm, 40 nm, and 63 nm Cu/Nb multi-layers demonstrating the effects of transverse (TRANS) and longitudinal (LONGL) loading directions plotted with solid and dash lines, respectively.

Emphasizing the size and geometrical effects, four thickness combinations of 34 nm and 63 nm along with another case with their uniform average thickness of 48.5 nm are simulated with otherwise identical settings. The outcomes are presented in Figure 5a where the strain rate of $10^{-3}$/s and the KS orientation relationships are considered.

As noted, the strongest pattern is the one with the smallest similar thicknesses signifying the predominant influence of size over the other traits. Among the rest, with a total thickness of 97 nm, the samples with the lower and higher thickness of niobium exhibit the strongest and weakest responses, respectively. The curve with the equal average thickness reveals a trend between the upper and lower bound, however, close to the latter. It is inferred that in cases of bilayers with two different crystal structures, one of the constituents has more influence on the overall mechanical properties than the other. Here, the effect of the body-centered cubic niobium with lower activation volumes is more decisive and almost twice as of the face-centered cubic copper on the whole responses either in the reduction or promotion of thicknesses. These effects are better recognized through the equivalent plastic strain defined as $E_{eq}^{p}=\sqrt{\frac{2}{3}\left(E^{p}:E^{p}\right)}$, where $E^{p}=\frac{1}{2}\left(F^{pT}F^{p}-I\right)$ and plotted in Figure 5b for each case. In the general trends and magnified region, the equivalent strain curves demonstrate the inverse relation with the layer strengths captured in Figure 5a and indicate the largest values for the weakest and smallest ones for the strongest case.

To investigate the load or layer direction effects, the simulations are performed for laminates of 34 nm, 40 nm, and 63 nm under both longitudinal and transverse loading directions, displayed in Figure 5c. Slight differences at the beginning stages of the plasticity are detected increasing with subsidence in layer spacing. However, the identical results in the extended plastic region demonstrate the inconsequential impacts of variations in the loading or layer orientation especially for detecting the flow strength of bilayers at the strain of about 10%.

**Figure 6:**
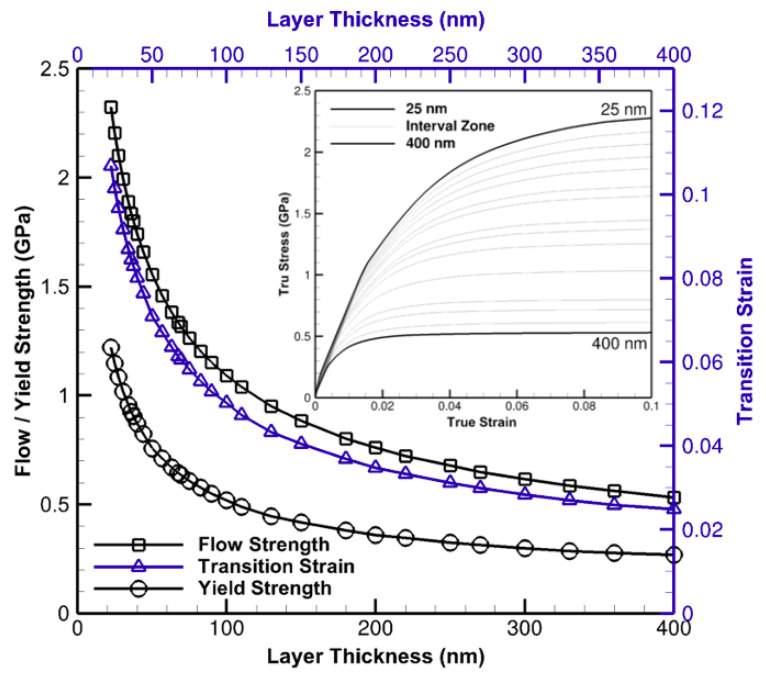
The variation of flow and yield strength (**left vertical axis**) as well as transition strain (**right vertical axis**), respectively, with respect to layer thickness in the range of 25 nm to 400 nm. The true stress-strain curves in this range is attached to the top right corner to clarify the overall constitutive behavior.

The significance of size effects is delineated in Figure 6 where the flow and yield strength as well as the transition strain in a wide nano-scale interval, 25 nm to 400 nm, are plotted. Considering the transition strain as the strain sustained from the yield to the onset of flow, a nonlinear descending trend of flow and yield strength is noted as layer spacings decline. Yield points are recognized when the resulted data from computational analyses start to deviate from the linear trend, albeit, with a tolerance consideration, and transition strains are detected once a hardening trend and the tangential line of a post-yield curve intersect. The increase in strain transition is primarily due to mechanical thresholds and dislocation structure evolutions aligned with low strain hardening and dynamic annihilation-recovery mechanisms. A small variation in thickness results a dramatic change in flow and yield strengths at the thicknesses of ⪅100 nm.

This bias has a descending followed by an asymptotic trend whilst the thickness approaches 1 μm. The similar trajectory in transition strain is indicative of an extended prehardening phenomenon pronounced especially at this range that continues to shrink and assume a higher curvature nearing 1 μm.

Although the developed models satisfyingly capture metallic nano-layers responses over a broad length scale, the time- and energy-consuming feature of the analysis is a hurdle to be overcome for which the multi-scale concept is sought and implemented for which effective functionals must be realized as discussed in the next section.

### 3.2. Homogenized Level Effective Functionals and Constants

**Figure 7:**
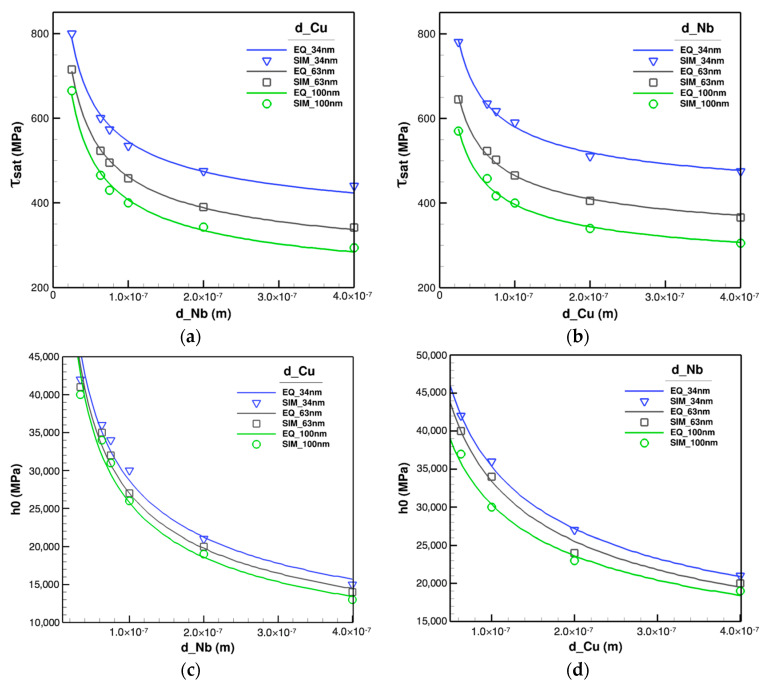
Variations of effective parameters in homogenized constitutive model with layer thicknesses where one layer thickness is fixed while the other one changes. Symbolic points signify simulation (SIM) results and solid lines the best fitted equivalent curves (EQ). Variations of $\tau_{\mathrm{sat}}$, for (**a**) fixed Cu layer spacing, d_Cu, and (**b**) fixed Nb layer spacing, d_Nb. Variations of $h_{0}$, for (**c**) fixed Cu layer spacing, d_Cu, and (**d**) fixed Nb layer spacing, d_Nb.

Sensitivity analyses detect two influential constitutive functionals to be calibrated from the lower scale; saturation shear stress, $\tau_{\mathrm{sat}}$, and initial hardening, $h_{0}$. These are functions of each constituent layer thickness, e.g., $\tau_{sat}\left(d_{Cu},d_{Nb}\right)$ and $h_{0}\left(d_{Cu},d_{Nb}\right)$ for Cu/Nb nano-layers. In order to obtain the relations of the effective functionals in terms of each material, several cases with different layer thicknesses of Cu and Nb are made. Two major sets of nano-layers are considered in which the thickness of one material is fixed at 34 nm, 63 nm, and 100 nm while the other one varied from 25 nm to 400 nm and vice versa. Then, the simulations are performed based on the size-dependent constitutive model at nano-scale regime, Equation (12), and processed through Hill–Mandel principle resulting in the calibration plots of $\tau_{\mathrm{sat}}$ and $h_{0}$ demonstrated in Figure 7.

The variation of $\tau_{\mathrm{sat}}$ with constant $d_{Cu}$ and varying $d_{Nb}$ is plotted in Figure 7a where the best fitted function for simulated data has the form of $\alpha_{1}+\frac{\alpha_{2}}{\sqrt{d_{Nb}}}$. The same process for Nb yields the similar functional structure with $\alpha_{3}+\frac{\alpha_{4}}{\sqrt{d_{Cu}}}$ plotted in Figure 7b. However, the best fitted functions for $h_{0}$ is different and has the format of $\beta_{1}+\frac{\beta_{2}}{\sqrt[{3}]{d_{Cu}}}$ when $d_{Nb}$ is fixed and $\beta_{3}+\frac{\beta_{4}}{\sqrt[{3}]{d_{Nb}}}$ while $d_{Cu}$ is constant as illustrated in Figure 7c,d, respectively. Consequently, the final formulations of the effective functionals are derived as

(29) $$\tau_{sat}=\left(\alpha_{1}+\frac{\alpha_{2}}{\sqrt{d_{Cu}}}\right)\left(\alpha_{3}+\frac{\alpha_{4}}{\sqrt{d_{Nb}}}\right)\;,$$

and

(30) $$h_{0}=\left(\beta_{1}+\frac{\beta_{2}}{\sqrt[{3}]{d_{Cu}}}\right)\left(\beta_{3}+\frac{\beta_{4}}{\sqrt[{3}]{d_{Nb}}}\right)\;,$$

for generalized circumstances when both $d_{Cu}$ and $d_{Nb}$ are changing. These equations have four unknowns to be determined. Due to the high nonlinearity of the acquired equations, ascertaining $\alpha_{i}\;,\;\beta_{i}$ necessitates a thorough, compatible optimization scheme. Thereby, a metaheuristic genetic algorithm approach is utilized to attain the parameters which results in the following equations.

(31) $$\tau_{sat}=\left(12.6169+\frac{0.0028}{\sqrt{d_{Cu}}}\right)\left(9.0473+\frac{0.0032}{\sqrt{d_{Nb}}}\right)\;,$$

and

(32) $$h_{0}=\left(48.3222+\frac{0.4358}{\sqrt[{3}]{d_{Cu}}}\right)\left(23.4275+\frac{0.7791}{\sqrt[{3}]{d_{Nb}}}\right)\;.$$

The homogenized constitutive model enhances the efficacy of computational processes in diverse aspects. Clarifying this matter, five random microstructures with different layer thicknesses are simulated; first, with the size-dependent constitutive model at nano-scale, Equation (12), and second, through the homogenized constitutive model, Equation (28), along with the realized effective functionals in Equations (31) and (32).

**Figure 8:**
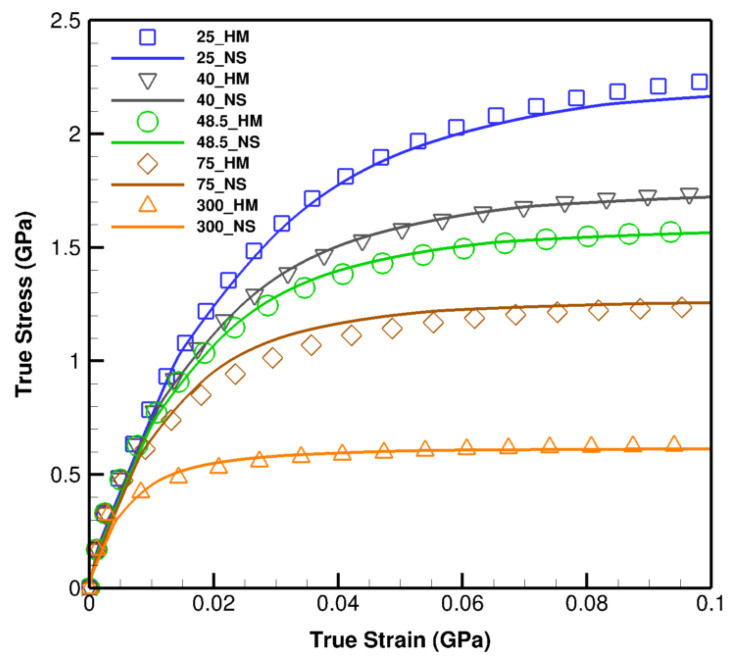
The comparison of the results obtained through the homogenized and nano-scale size-dependent constitutive model on 25 nm, 40 nm, 48.5 nm, 75 nm, and 300 nm Cu/Nb laminates. Symbolic points denote homogenized (HM) and solid lines the nano-scale (NS) model results.

The nano-scale simulations proceed through the model with the explicit representation of layer thickness while the homogenized model is executed by the implicit impact of size rendered through effective functionals in Equations (31) and (32). The results and comparisons shown in Figure 8 exhibit cogent agreements between two models, albeit, the homogenized constitutive model significantly reduces the computational time and cost by several (>4) orders of magnitude.

### 3.3. Homogenized Level Temperature Effects

In general, the elevated temperature induces relative diffusive mass flux due to energy gradients. Diffusional creep is considered the main deformation mechanism at the vicinity of the melting point, $T_{m}$, in metallic nano-layers [53] where the stress-driven diffusion of vacancies along grain boundaries compels atomic diffusion of the grain interiors in the opposite direction. This effect is alleviated by atomic diffusion along grain boundaries at lower temperature [54] while the dislocation glide along grain boundaries becomes the dominant mechanism at intermediate and low homologous temperatures. Cu/Nb cases, at temperatures up to 800 °C, exhibit dislocation-based plastic deformation where diffusion creep can be ignored due to generated thermally stable structures [55,56]. Being cognizant of the experimental difficulties mentioned in Section 1 for obtaining mechanical responses of metallic nano-layers at elevated temperatures, a temperature-dependent constitutive model is developed with the advantages of acquiring responses through fast and cost-effective performances.

The homogenized constitutive model in Equation (28) works with a mild variation of ambient temperature; however, generic temperature variations require additional changes in some of the material constants and constitutive parameters.

The elastic constants can be written as a function of absolute temperature by $C_{ij}=\chi_{ij}+\omega_{ij}T$ [57] and shear modulus with $\mu =m_{1}+m_{2}T$, where the constants, $\chi_{ij}\;,\;\omega_{ij}\;,\;m_{1}\;,\;m_{2}$ are designated in Table 4 for Cu/Nb nano-layers.

The effective functionals, saturation shear resistance and initial hardening, also change in terms of temperature. To achieve the general format of these functionals and obtain the associated parameters, experimental data in [49,55] are incorporated through the deep-learning SLC and metaheuristic genetic algorithms. As a result, effective temperature-dependent functionals are obtained as

(33) $$\tau_{sat}=\left[\psi_{0}\exp\left(\frac{\zeta }{T-T_{c}}\right)+\psi_{1}\right]\left(12.6169+\frac{0.0028}{\sqrt{d_{Cu}}}\right)\left(9.0473+\frac{0.0032}{\sqrt{d_{Nb}}}\right)\;,$$

and

(34) $$h_{0}=\left(\eta_{0}+\eta_{1}T\right)\left(48.3222+\frac{0.4358}{\sqrt[{3}]{d_{Cu}}}\right)\left(23.4275+\frac{0.7791}{\sqrt[{3}]{d_{Nb}}}\right)\;,$$

where the associated parameters of $\psi_{0}\;,\;\psi_{1}\;,\;\zeta \;,\;T_{c}\;,\;\eta_{0}$, and $\eta_{1}$ are calibrated as shown in Table 5.

**Figure 9:**
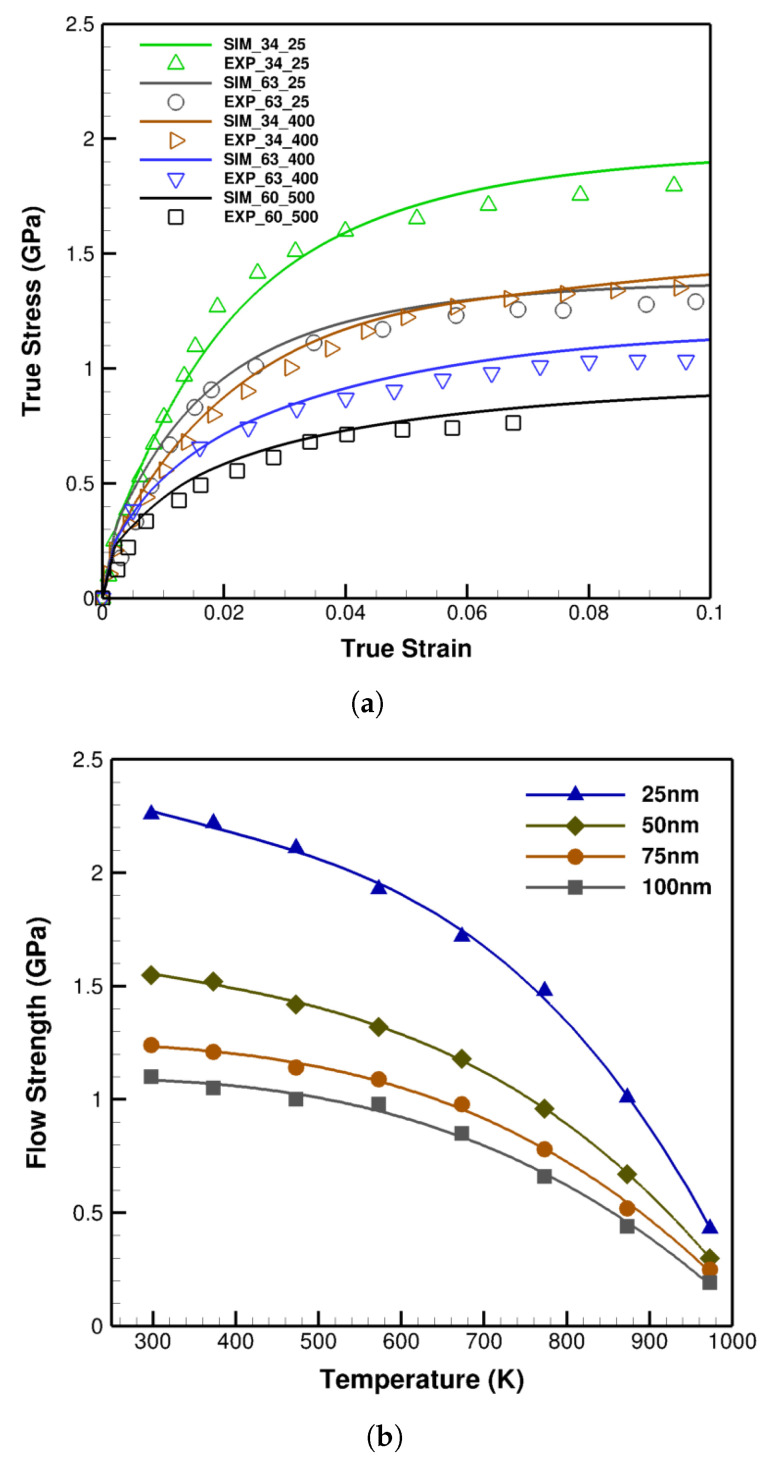
(**a**) The validation of the temperature-dependent constitutive model with 34 nm, 60 nm, and 63 nm Cu/Nb laminates at 25 °C, 400 °C, and 500 °C. Symbolic points are the experimental (EXP) [49,55] and solid lines the simulation (SIM) data. (**b**) Flow strength versus temperature curves of 25 nm, 50 nm, 75 nm, and 100 nm Cu/Nb laminates at 25 °C up to 700 °C demonstrating the nonlinear effects of temperature growth on flow strength.

For verification, simulations are performed for Cu/Nb multi-layers with thicknesses of 34 nm, 60 nm, and 63 nm at 25 °C, 400 °C, and 500 °C as demonstrated in Figure 9a. As observed, the simulations and experimental results exhibit solid agreements in which dramatic declines in flow stresses by increasing temperature are plainly detected.

Further illuminating this phenomenon, Cu/Nb multi-layers with 25 nm, 50 nm, 75 nm, and 100 nm thicknesses are modeled from room temperature up to 700 °C where the variation of flow strengths in terms of temperature is displayed in Figure 9b. Each curve is indicative of slight variation in flow stress at initial stages while revealing an appreciable drop as temperature grows. For instance, in 25 nm specimen, the flow stress notably, about 80%, drops from room temperature to 700 °C.

From another angle, the increase of temperature degrades the mechanical responses of a thin metallic nano-layer to a thicker one at room temperature; this can be clearly perceived in Figure 9a where a 34 nm Cu/Nb at 400 °C exhibits the strength of a 63 nm Cu/Nb at 25 °C.

## 4. Conclusions

The current work develops multi-scale constitutive models and deep-learning SLC approaches in two major scales of the nano- and homogenized levels. CPFE in the large deformation platform was utilized to reflect the anisotropic and rate-dependent nature of the metallic nano-systems, simulate, and predict associated responses where Cu/Nb nano-layers as case studies were incorporated in diverse three-dimensional thermomechanical loading conditions.

At the nano-scale, a size-dependent constitutive model founded on entropic kinetics was developed with the explicit size and constituent effects along with hardening evolution. The SLC as a deep-learning adaptive boosting technique was established to acquire generalized constitutive parameters through a single process while remaining applicable to a broad scope of settings regardless of any difference with the calibration setups. The models were validated through experimental results and utilized for further behavioral prediction in terms of size, loading pattern, layer type, and geometrical effects where size and constituent effects were plainly captured on flow strength and transition strain.

At the homogenized scale, statistical analyses were employed to develop a homogenized crystal plasticity-based constitutive model for expediting the computational process. The elastic constants and effective functionals were realized and associated parameters obtained via metaheuristic genetic algorithms. The homogenized responses were solidly verified with nano-scale data while the computational processes were accelerated by several orders of magnitude.

A temperature-dependent homogenized constitutive model was developed for which elastic constants and effective functionals were constructed. The related constants were obtained and the model was favorably validated with experimental data. Ultimately, the nonlinear effects of temperature on flow strength for several cases were predicted, analyzed, and discussed.

## Figures and Tables

**Table 1:** Material parameters to be determined from a deep-learning method and experimental data.

Constitutive Model Parameters									
${\dot{\gamma }}_{0}^{\alpha }$	*p*	*q*	*r*	$c_{s}$	$c_{sat}$	*m*	$\tau_{\mathrm{cut}}^{\alpha }$	$\tau_{\mathrm{pass}-\mathrm{init}}^{\alpha }$	$h_{0}^{\beta }$

**Table 2:** Material constants of copper and niobium acquired from analytical processes and databases.

Material Elastic Constants	Cu	Nb
$C_{11}$(GPa)	168.4	246.0
$C_{12}$(GPa)	121.4	134.0
$C_{44}$(GPa)	75.4	28.7
μ(GPa)	48.0	38.0
$Q_{active}$(J)	$8.05\times 10^{-19}$	$8.9\times 10^{-19}$
*b*(m)	$2.56\times 10^{-10}$	$2.86\times 10^{-10}$

**Table 3:** Material parameters of copper and niobium acquired from the deep-learning SLC and experimental data.

Material Parameters	Cu	Nb
${\dot{\gamma }}_{0}^{\alpha }$	5.0 × 10^6^	6.0 × 10^6^
*p*	0.98	1.01
*q*	1.12	1.14
*r*	1.115	1.121
$c_{s}$	0.2	0.002
$c_{sat}$	70,000.0	76,741.0
*m*	−0.50	−0.50
$\tau_{\mathrm{cut}}^{\alpha }$ (MPa)	4.80	6.30
$\tau_{\mathrm{pass}-\mathrm{init}}^{\alpha }$ (MPa)	5.20	7.10
$h_{0}^{\beta }$ (MPa)	3000.0	20,000.0

**Table 4:** Elastic parameters of copper and niobium acquired from the calibration process.

Cu				Nb			
$\chi_{11}$(GPa)	184.13	$\omega_{11}$(GPa/K)	−0.05	$\chi_{11}$(GPa)	262.70	$\omega_{11}$(GPa/K)	−0.06
$\chi_{12}$(GPa)	133.32	$\omega_{12}$(GPa/K)	−0.04	$\chi_{12}$(GPa)	143.33	$\omega_{12}$(GPa/K)	−0.03
$\chi_{44}$(GPa)	88.15	$\omega_{44}$(GPa/K)	−0.04	$\chi_{44}$(GPa)	40.18	$\omega_{44}$(GPa/K)	−0.01
$m_{1}$(GPa)	52.95	$m_{2}$(GPa/K)	−0.02	$m_{1}$(GPa)	30.88	$m_{2}$(GPa/K)	−0.01

**Table 5:** Saturation shear resistance and initial hardening parameters.

$\psi_{0}$	$\psi_{1}$	ζ	$T_{c}$	$\eta_{0}$	$\eta_{1}$
7.31	−5.72	100.00	1450.00	1.42	−0.0014

## Data Availability

The data that support the findings of this study can be accommodated upon reasonable request.

## Appendix A. General Steps of Solving Equilibrium Equation—FE

1. Equilibrium Equation $\int_{v}\sigma :\delta e\;dv-\int_{\Gamma }t\;\delta v\;d\Gamma =0$
2. Finite Element Discretization
  $\delta e=\frac{1}{2}\left(\delta l+\delta l^{T}\right)$
  $v=\sum_{i=1}^{nNode}N_{i}\hat{v_{i}}$ $\delta l=\frac{\partial \delta v}{\partial x}=\sum_{i=1}^{nNode}\hat{v_{i}}⊗\nabla_{x}N_{i}=\nabla_{\left(\xi ,\eta ,\zeta \right)}N_{i}\left[\sum_{i=1}^{nNode}x_{i}⊗\nabla_{\left(\xi ,\eta ,\zeta \right)}N_{i}\right]\hat{v}$
3. Residual Force
  $R\left(\hat{v}\right)=\int_{v}{\left(\nabla_{x}N_{i}\right)}^{T}\sigma dv-\int_{s}N_{i}t\delta d\Gamma =0$
4. Newton-Raphson Solver
  ${\hat{v}}^{n+1}={\hat{v}}^{n}-{\left(\frac{\partial R}{\partial \hat{v}}\right)}_{n}^{-1}R_{n}$
5. Residual Derivative
  $\frac{\partial R}{\partial \hat{v}}=\int_{v}{\left(\nabla_{x}N_{i}\right)}^{T}k\left(\nabla_{x}N_{i}\right)dv+\int_{v}{\left(\nabla_{x}N_{i}\right)}^{T}\sigma ⊗\left(\nabla_{x}N_{i}\right)dv$
6. Material Stiffness
  $K_{M}=\int_{v}{\left(\nabla_{x}N_{i}\right)}^{T}k\left(\nabla_{x}N_{i}\right)dv=\int_{v}B^{T}D_{ep}Bdv$
7. Geometrical Stiffness
  $K_{\sigma }=\int_{v}{\left(\nabla_{x}N_{i}\right)}^{T}\sigma ⊗\left(\nabla_{x}N_{i}\right)dv=\int_{v}B_{\sigma }^{T}\sigma B_{\sigma }dv$
8. Calculation of σ and $D_{ep}$ in Appendix B.

## Appendix B. General Steps of Acquiring System Stiffness—CP

1. Kinematics
  $F\left(\tau \right)=F^{e}\left(\tau \right)\;F^{p}\left(\tau \right)\;,\;\dot{F^{p}}\left(\tau \right)=l^{p}\left(\tau \right)\;F^{p}\left(\tau \right)$
2. Plastic Deformation Rate Dependence
  $l^{p}=\sum_{\alpha =1}^{nslip}{\dot{\gamma }}^{\alpha }\left(\tau \right)\;m_{0}^{\alpha }⊗n_{0}^{\alpha }\;\Rightarrow \;\dot{F^{p}}=\left(\sum_{\alpha =1}^{nslip}{\dot{\gamma }}^{\alpha }\left(\tau \right)\;m_{0}^{\alpha }⊗n_{0}^{\alpha }\right)F^{p}$
3. Second Piola–Kirchhoff Stress
  $S\left(\tau \right)=\frac{1}{2}C\left({F^{e}}^{T}\left(\tau \right)F^{e}\left(\tau \right)-I\right)$
  $$S\left(\tau \right)=\frac{C}{2}\left[\sum_{\alpha =1}^{nslip}\left(I-\Delta \gamma^{\alpha }\;{\left(m_{0}^{\alpha }⊗n_{0}^{\alpha }\right)}^{T}\right){F^{p}}^{-T}\left(t\right)F^{T}\left(\tau \right)F\left(\tau \right)F^{-p}\left(t\right)\sum_{\alpha =1}^{nslip}\left(I-\Delta \gamma^{\alpha }\;m_{0}^{\alpha }⊗n_{0}^{\alpha }\right)-I\right]$$
4. Elastoplastic Parts
  $S_{el}\left(\tau \right)=\frac{C}{2}\left({F^{p}}^{-T}\left(t\right)F^{T}\left(\tau \right)F\left(\tau \right)F^{-p}\left(t\right)-I\right)$
  $S_{pl}\left(\tau \right)=-\frac{C}{2}\left({F^{p}}^{-T}\left(t\right)F^{T}\left(\tau \right)F\left(\tau \right)F^{-p}\left(t\right)\sum_{\alpha =1}^{nslip}\left(\Delta \gamma^{\alpha }\;m_{0}^{\alpha }⊗n_{0}^{\alpha }\right)\right)\;-\frac{C}{2}\left(\sum_{\alpha =1}^{nslip}{\left(\Delta \gamma^{\alpha }\;m_{0}^{\alpha }⊗n_{0}^{\alpha }\right)}^{T}{F^{p}}^{-T}\left(t\right)F^{T}\left(\tau \right)F\left(\tau \right)F^{-p}\left(t\right)\right)$
5. Nonlinear Solution–Defined Residual Function
  $G\left(S\right)=S\left(\tau \right)-S_{el}-\frac{C}{2}\sum_{\alpha =1}^{nslip}F\left(\alpha \right)\Delta \gamma^{\alpha }$
6. Nonlinear Iteration Obtaining 2nd Piola–Kirchhoff Stress
  $S^{\left(i+1\right)}=S^{\left(i\right)}-J^{-1}\left[S^{\left(i\right)}-S_{tr}+\sum_{\alpha =1}^{nslip}F\left(\alpha \right)\;\Delta \gamma^{\alpha }\right]$
  $J=I+\sum_{\alpha =1}^{nslip}F\left(\alpha \right)⊗\;\frac{\partial \gamma^{\alpha }}{\partial S}$
7. Updated Constitutive Model and Evolving Parameters
  ${\dot{\gamma }}^{\alpha }={\dot{\gamma }}_{0}^{\alpha }\exp\left\{-\frac{Q_{active}}{K_{B}T}{\left[1-{\left(\frac{{\left(\tau_{\mathrm{eff}}^{\alpha }\right)}^{2}}{\tau_{\mathrm{cut}}^{\alpha }}\frac{c_{s}\pi }{\mu b}\;d\right)}^{p}\right]}^{q}\right\}\mathrm{sgn}\left(\tau^{\alpha }\right)$
  ${\dot{\tau }}_{pass}^{\alpha }=\sum_{\beta =1}^{nslip}h^{\alpha \beta }\left|{\dot{\gamma }}^{\beta }\right|$
8. Elastic Deformation Gradient
  $\left(\mathrm{Converged}\;\;\sigma \;,\;F^{p}\right)$ $\to F^{e}\left(\tau \right)=F\left(\tau \right)F^{-p}\left(\tau \right)$
9. Cauchy Stress
  $\sigma \left(\tau \right)=\frac{1}{\det F^{e}\left(\tau \right)}{F^{e}}^{T}\left(\tau \right)S\left(\tau \right)F^{e}\left(\tau \right)$
10. Elastoplastic Material Tensor
  $D_{ep}↓=W=\frac{\partial \sigma }{\partial E}$
